# Another Turn of the Page

**DOI:** 10.1093/asj/sjad278

**Published:** 2023-11-16

**Authors:** Foad Nahai

It was an honor, with much excitement and anticipation, that on January 1, 2009 I assumed the editorship of *Aesthetic Surgery Journal* and wrote my first editorial, “Turning the Page.”^1^ In that first editorial I made the following observations:

“It is inevitable that changes in healthcare—and perhaps more ups and downs in the economy—will challenge and impact all of us in the years ahead. Whatever changes come, however, we can be certain that cosmetic surgery and medicine will continue to progress at an almost overwhelmingly rapid pace. It is also clear that an increasing number of physicians and nonphysicians will enter the field. The *Journal* is well positioned to stay at the forefront of clinical, scientific, and technological advances and to bring you the information you need to stay ahead of the curve. That goal, along with our commitment to patient safety, is our most important mission.”

Yes, much has changed over the past 15 years, but as I turn another page, those words are as true now as they were then.

Some changes that impact the *Journal* are under our own control, but those pale when compared with changes we cannot control. The world around us is continually changing, and the economy remains consistent only in its cyclical ups and downs, which directly affect aesthetic surgery spending. Despite the economic cycles, interest in aesthetic surgery and cosmetic medicine continues at an impressive pace. Innovations in treatment and the development of new drugs and devices are keeping pace with the demand. Our commitment to be at the forefront of these developments, bringing evidence-based information to our readers and promoting the best interests of patient safety, remains the core mission of the *Journal* and that is well within our control. Decisions on what we publish, how often we publish, who serves on our editorial board, and who serves as a reviewer are all the responsibility of the editorial team.

The economy, the changing landscape of scholarly publishing, the predicted end of printed journals, the continued growth of online-only publishing, the rise of open-access journals, and most recently the disruptive forces of artificial intelligence are all beyond our control, but we have the will and the ability to adapt to and accept the changes affecting us today by embracing them for the *Journal*'s benefit now and beyond 2023.

My intent in this final editorial as editor-in-chief is not to chronicle the success and continued rise of *ASJ*, its Impact Factor, or the rising circulation and international reach over the last 15 years. The credit for any success we have enjoyed goes to the team with whom I have been fortunate enough to work. The disappointments, which fortunately were very few, are my responsibility.

I have always maintained the philosophy that the hallmark of any scholarly publication is to present divergent views and to promote debate and discussion in a civil and respectful manner. Central to our belief has always been that regardless of our success and international ranking, complacency and arrogance would remain alien to us.

It remains for me to express my gratitude to those who have made my tenure a joy and those who shared my “labor of love” commitment to a “24/7, 8 days a week” work ethic. In January 2009, the team consisted of Melissa Berbusse Schmidt, who served as executive editor, and me. As we grew, Hunter Alexander joined the team, serving as managing editor for many years. Next, Phaedra Cress was hired as executive editor, now serving as executive publisher, journals; followed by Kyleigh Vrettos, editorial associate; Abby Pugh, managing editor of *ASJ*; Laura Simson, managing editor of *ASJ Open Forum*; and Anastasia Cyzewski, assistant managing editor of *ASJ*. To this team and the others who served along the way, I offer my sincere thanks and appreciation for your tireless commitment, enthusiasm, and contributions.

During my tenure, I have had the privilege of working with 15 presidents and boards of directors of The Aesthetic Society. The boards allowed me full editorial freedom. Both the journals and I benefited from their support and that of the Society staff, led by Executive Director Sue Dykema, and Bob Stanton before her. It has been a pleasure to work with our section editors, editorial board, reviewers, and authors. Thank you. I have learned much from all of you.

The page turns to my good friend, *ASJ*'s associate editor and *ASJ Open Forum*'s editor-in-chief, Dr Jeffrey Kenkel, who has been by my side for 15 years. He is a scholar, a researcher, a leader, a contributor, an outstanding surgeon, and most of all, an accomplished educator. Given his many qualifications, I have no doubt that the *Journal* is in the best of hands and that it will continue to enjoy many successes in the years to come.

As editor I had the privilege and pleasure of writing editorials on a regular basis.^2-6^ That, I shall miss. It was an honor back in 2009 to be entrusted with the editorship of *Aesthetic Surgery Journal*, and I am honored to have served the *Journal*, The Aesthetic Society, our specialty, and our readers.

My term with the *Journal* is coming to an end, but this is not a retirement. I plan to continue the full practice of aesthetic surgery, and to continue to contribute to the specialty I love—and yes, I will be watching *ASJ* from afar.
